# Weight loss–independent associations of body composition changes with device-derived metabolic age during Mediterranean diet follow-up

**DOI:** 10.1590/1806-9282.20260845

**Published:** 2026-10-05

**Authors:** Ahmet Emre Hatır, Mustafa Onmaz, Reyhan Vildan Acar

**Affiliations:** 1 Bandırma Onyedi Eylul University, Department of Family Medicine – Balıkesir, Türkiye.; 2 Hasan Katırcı Healthy Living Center – Konya, Türkiye.

**Keywords:** Body composition, Electric impedance, Mediterranean diet, Visceral fat

## Abstract

**OBJECTIVE:**

The aim of this study was to investigate whether changes in body composition over 6 months of Mediterranean diet follow-up were associated with corresponding changes in device-derived metabolic age after adjustment for weight change.

**METHODS:**

This retrospective observational study analyzed body composition records of 414 adults who completed routine 6-month follow-up after Mediterranean diet counseling between June 2024 and June 2025. Metabolic age and body composition parameters were assessed using the Tanita MC-580 body composition analyzer at baseline and after 6 months. Hierarchical multiple linear regression analysis was used to evaluate the associations between changes in body composition parameters and metabolic age change after adjustment for weight change, age, sex, and baseline metabolic age.

**RESULTS:**

Tanita-derived metabolic age decreased by 0.94 years (p<0.001). Changes in muscle–fat ratio (β=-0.585), visceral fat (β=-0.556), and protein mass (β=-0.339) were significantly associated with metabolic age change after adjustment, whereas weight-adjusted waist index was not significantly associated with metabolic age change in the adjusted model. Inclusion of body composition variables increased the explained variance of the model from 20.4 to 45.7% (adjusted R^2^ from 0.196 to 0.446).

**CONCLUSION:**

Favorable changes in Tanita-derived metabolic age were observed during follow-up. The associated body composition changes may partly reflect shared components of the device algorithm because metabolic age is a composite surrogate rather than a direct measure of biological aging. Given the retrospective uncontrolled design, causal effects of the Mediterranean diet cannot be established.

## INTRODUCTION

Metabolic age is often used as a simple indicator of overall physical and metabolic condition. It is estimated by comparing an individual’s basal metabolic rate (BMR) with the expected BMR for people of the same chronological age. In general, a lower metabolic age is thought to be associated with a more favorable cardiometabolic profile. However, this measure should be interpreted cautiously, as it is a device-derived estimate shaped by body composition and related physiological features rather than a direct biological marker. Factors such as genetics, muscle and fat mass, physical activity, and dietary habits may all influence metabolic age^1,2^. Among dietary approaches, the Mediterranean diet has received particular attention because of its associations with improvements in body weight, lipid profile, blood pressure, and insulin sensitivity^3^. Accordingly, changes in this device-derived estimate during dietary follow-up may coincide with both weight change and changes in body composition.

Body composition, especially the balance between fat mass and lean tissue, plays an important role in metabolic health. Visceral fat is closely linked to insulin resistance, dyslipidemia, and higher cardiovascular risk, even in individuals without marked excess body weight. In contrast, a greater proportion of lean mass is generally associated with better metabolic regulation and physical performance. Evidence from recent studies suggests that dietary interventions can improve metabolic outcomes by reducing harmful fat depots, particularly visceral fat, even when total weight loss is relatively modest^3,4^. Beyond these major body compartments, body water compartments are also measured by bioelectrical impedance analysis (BIA) and may vary with tissue composition and fluid distribution^5,6^.

Although metabolic age is increasingly used as a simple device-derived indicator in body composition assessment, its clinical interpretation after dietary follow-up remains insufficiently defined. In routine practice, dietary response is often evaluated primarily through body weight, body mass index (BMI), and waist circumference, whereas these parameters may not fully capture qualitative changes in body composition. Importantly, because device-derived metabolic age is calculated using BMR and body-composition-related inputs, associations between this estimate and BIA-derived variables may partly reflect shared algorithmic components. Nevertheless, examining these adjusted associations may help clarify how the device-derived estimate behaves during routine follow-up. Therefore, the present study aimed to examine the associations between changes in body composition parameters and changes in Tanita-derived metabolic age during 6-month Mediterranean diet follow-up after accounting for weight change and baseline covariates.

## METHODS

This retrospective observational study was based on the analysis of body composition records from individuals who participated in a Mediterranean diet follow-up between June 2024 and June 2025. Measurements obtained at baseline (month 0) and at 6 months were analyzed. Participants were not prospectively randomized or allocated by the investigators for research purposes; rather, routinely collected records of individuals who had attended a dietitian-led Mediterranean diet follow-up program were analyzed. During the study period, all available records of individuals attending the Mediterranean diet follow-up were screened sequentially. Of 465 baseline records screened, 51 were excluded because of missing 6-month measurements, missing or erroneous data, or predefined exclusion criteria. The final complete-case cohort included 414 participants, representing 89.0% of the screened baseline records. Because this was a retrospective study based on routinely collected records, no a priori sample size calculation was performed. Instead, all eligible participants with complete baseline and 6-month measurements during the predefined study period were included. Participants followed a nutritional program based on Mediterranean diet principles under the guidance of a registered dietitian. Dietary counseling emphasized increased consumption of vegetables, fruits, legumes, whole grains, olive oil, nuts, and fish, together with reduced intake of refined carbohydrates, processed foods, sugar-sweetened products, red and processed meats, and saturated fat-rich foods. Adherence was assessed during routine dietitian-led follow-up visits based on the dietary information documented in the clinical records; no validated Mediterranean diet adherence scale, dietary recall, food diary, food-frequency questionnaire, or objective nutritional biomarker was used. Changes in participants’ physical activity habits and medication use were not systematically monitored. Chronic disease status and laboratory test results were not incorporated into the analyses. Participants were eligible if they were aged 18 years or older, had undergone multi-frequency BIA at both time points, had documented follow-up for 6 months, and met the pre-measurement fasting requirement of 8–12 h. Individuals were excluded if they had a history of acute infection, malignancy, renal or hepatic failure, or other systemic diseases; were pregnant or breastfeeding; had pacemakers or metallic implants; had severe edema or fluid imbalance; had incomplete or erroneous follow-up records; or were younger than 18 years of age.

Body composition was measured using a segmental bioelectrical impedance analyzer (Tanita MC-580; Tanita Corporation, Tokyo, Japan), which employs an eight-electrode hand-to-foot system and dual-frequency BIA (6.25 and 50 kHz)^7,8,9^. All measurements were taken in the morning after fasting for 8 to 12 h. Participants were asked to avoid vigorous physical activity in the past 24 h, empty their bladder, and remove metallic accessories prior to the test. Room temperature was maintained between 22 and 24°C. Age, height, and sex were entered into the device before measurement. The analyzer was calibrated according to the manufacturer’s instructions, and all measurements were performed by the same operator. BMI was computed as weight in kilograms divided by height in meters squared. Weight-adjusted waist index (WWI) was computed by dividing waist circumference in centimeters by the square root of body weight in kilograms. Muscle-to-fat ratio (MFR) was defined as skeletal muscle mass divided by fat mass. Metabolic age is a proprietary composite surrogate estimate derived by the device from BMR and body-composition-related parameters, including fat-free mass and body fluid distribution. It should therefore be interpreted as a device-derived output rather than as a direct measure of biological or epigenetic aging processes^9^.

The study was conducted in accordance with the Declaration of Helsinki and the national ethical standards of Turkey, following ethical approval from the Ethics Committee for Non-Interventional Clinical Research at KTO Karatay University, Faculty of Medicine (Approval No: 2026/032, protocol number 132293, date: 26 February 2026).

### Statistical analysis

Data were analyzed using International Business Machines Statistical Package for the Social Sciences Statistics for Windows, version 27.0 (IBM Corp., Armonk, NY, USA). Figure 1 was generated using Python (version 3.10). Even though normality tests were statistically significant, skewness and kurtosis were within acceptable limits; parametric analyses were therefore considered robust. Continuous variables were presented as mean±standard deviation. The primary outcome was metabolic age change, calculated as the 6-month value minus the baseline value (Δ). Paired comparisons were performed using the paired samples t-test. Hierarchical multiple linear regression analysis was used with metabolic age change as the dependent variable. For each regression model, unstandardized coefficients (B), standard errors (SEs), standardized coefficients (β), 95%CIs, p-values, and variance inflation factor (VIF) values were reported. Multicollinearity was assessed using VIF values. In the base model, weight change, age, sex, and baseline metabolic age were included. In Model 2, changes in protein mass, MFR, WWI, and visceral fat were added to evaluate their associations beyond weight change and baseline covariates. Candidate variables were selected based on clinical and physiological relevance, avoiding overfitting and multicollinearity^10^. A sensitivity power analysis was conducted using G*Power version 3.1 to determine the minimum incremental effect size detectable with the available sample in the hierarchical multiple regression analysis. With 414 participants, an alpha level of 0.05, four tested predictors, eight total predictors, and 80% statistical power, the minimum detectable incremental effect size was f^2^=0.029, corresponding to a small effect. The significance level was set at p<0.05.

## RESULTS

A total of 414 individuals were included. Participants had a mean age of 40.78±10.86 years (range 19–70). The study population comprised 342 women (82.6%) and 72 men (17.4%).

Significant changes were observed in metabolic age and most body composition parameters during follow-up. Metabolic age decreased significantly (-0.94 years, p<0.001). Substantial reductions were noted in body weight (-2.60 kg, p<0.001) and BMI (-1.02 kg/m^2^, p<0.001). Waist circumference, fat mass, visceral fat, and muscle mass indicators also changed significantly during follow-up (all p<0.001). MFR improved significantly, reflecting a more favorable lean-to-fat balance (p<0.001). WWI decreased significantly from 10.90±0.79 to 10.76±0.80 (Δ=-0.14, p<0.001), indicating an improvement in weight-adjusted central adiposity. Total body water showed a modest but significant decrease (p<0.001) (Table 1).

**Table 1.** Changes in anthropometric and body composition parameters during Mediterranean diet follow-up.

Variable	Month 0 Mean±SD	Month 6 Mean±SD	Mean Change (Δ)	p
Metabolic age (years)	47.81±11.69	46.86±11.93	-0.94	<0.001
Weight (kg)	83.25±17.21	80.65±16.65	-2.60	<0.001
BMI (kg/m^2^)	32.49±6.08	31.46±5.94	-1.02	<0.001
Waist circumference (cm)	99.52±17.04	96.69±16.72	-2.83	<0.001
Fat mass (kg)	31.29±11.07	29.39±10.92	-1.90	<0.001
Muscle mass (kg)	49.26±7.80	48.59±7.62	-0.67	<0.001
Skeletal muscle mass (kg)	29.42±4.68	29.02±4.57	-0.40	<0.001
Protein mass (kg)	12.01±2.93	11.95±2.80	-0.06	0.267
Mineral mass (kg)	3.65±0.63	3.60±0.61	-0.05	<0.001
Muscle–fat ratio	1.02±0.30	1.09±0.35	+0.07	<0.001
Weight-adjusted waist index	10.90±0.79	10.76±0.80	-0.14	<0.001
Visceral fat	8.64±4.13	8.02±4.02	-0.62	<0.001
Total body water (kg)	36.31±5.64	35.72±5.57	-0.59	<0.001

Changes in anthropometric and body composition parameters during 6-month Mediterranean diet follow-up. Values are presented as mean±standard deviation (SD). Mean change (Δ) represents the difference between Month 6 and baseline measurements. p-values were obtained using paired statistical tests. BMI: body mass index.

Hierarchical multiple linear regression analysis was performed to evaluate factors associated with metabolic age change after accounting for weight change and baseline covariates. In the base model (Model 1), which included weight change, age, sex, and baseline metabolic age, the model explained 20.4% of the variance in metabolic age change (adjusted R^2^=0.196, p<0.001). Weight change (β=0.459, p<0.001) and baseline metabolic age (β=0.269, p=0.032) showed statistically significant associations with metabolic age change. When body composition markers were added in Model 2, the explained variance increased from 20.4 to 45.7% (adjusted R^2^=0.446; ΔR^2^=0.253, p<0.001). Changes in muscle–fat ratio (β=-0.585, p<0.001), visceral fat (β=-0.556, p<0.001), and protein mass (β=-0.339, p<0.001) remained significantly associated with metabolic age change after adjustment. ΔWWI was not statistically significant (β=-0.135, p=0.124) (Table 2). The incremental explained variance across hierarchical regression models is illustrated in Figure 1.

**Table 2.** Hierarchical multiple linear regression analysis of factors associated with Δmetabolic age.

	Variables	B	SE	^β^	95%CI (LL–UL)	p	VIF	Adj. R^2^
Model 1 Base model (weight change controlled)	ΔWeight	0.321	0.032	0.459	0.259–0.384	<0.001	1.060	0.196
	Age	-0.029	0.021	-0.175	-0.069–0.012	0.161	7.964	
	Sex	0.120	0.212	0.025	-0.297–0.537	0.572	1.033	
	Baseline metabolic age	0.041	0.019	0.269	0.004–0.079	0.032	8.063	
Model 2 (+body composition)	ΔProtein	-0.522	0.099	-0.339	-0.717 to -0.327	<0.001	3.096	0.446
	ΔMuscle–fat ratio	-7.544	0.959	-0.585	-9.429 to -5.659	<0.001	4.123	
	ΔWeight-adjusted waist index	-0.957	0.621	-0.135	-2.178 to 0.264	0.124	5.729	
	ΔVisceral fat	-0.935	0.121	-0.556	-1.173 to -0.698	<0.001	3.843	

B represents the unstandardized regression coefficient; β represents the standardized coefficient. SE: standard error; CI: confidence interval; VIF: variance inflation factor. Model 1 included weight change, age, sex, and baseline metabolic age. Model 2 additionally included changes in protein mass, muscle–fat ratio, weight-adjusted waist index, and visceral fat. LL: lower limit; UL: upper limit.

**Figure 1:**
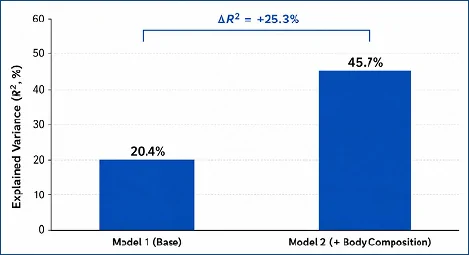
Incremental explained variance across hierarchical regression models. Explained variance across hierarchical regression models for change in device-derived metabolic age. Model 1 included baseline covariates and weight change. Model 2 additionally incorporated body composition variables, increasing the explained variance from R^2^=0.204 to R^2^=0.457 (ΔR^2^=0.253).

## DISCUSSION

During 6 months of Mediterranean diet follow-up, changes in MFR, visceral fat, and protein mass remained significantly associated with changes in Tanita-derived metabolic age after adjustment for weight change and baseline covariates. These findings are consistent with previous studies reporting favorable changes in body composition, fat distribution, and metabolic risk profiles during Mediterranean diet interventions^4,11^. Broader literature shows that adherence to a Mediterranean diet is associated with reduced central adiposity, improved metabolic syndrome markers, and lower cardiovascular risk^11,12,13,14,15^, and that combined lifestyle interventions including the Mediterranean diet improve visceral adiposity while preserving lean mass^4^. Importantly, the associations between BIA-derived body composition variables and changes in Tanita-derived metabolic age may partly reflect shared measurement and algorithmic components. The regression findings should therefore be interpreted as adjusted associations or correlates of change in the device-derived estimate, rather than as evidence that these variables independently determine metabolic or biological aging.

MFR change showed the largest standardized association with metabolic age change among the body composition variables included in Model 2. This is physiologically plausible because skeletal muscle plays a central role in glucose utilization, insulin sensitivity, and resting energy expenditure, whereas excess adiposity is associated with chronic low-grade inflammation^16,17^. Nevertheless, because MFR and metabolic age were both derived from the same BIA assessment, this association should be interpreted cautiously and may partly reflect shared algorithmic components. The adjusted association between visceral fat change and metabolic age change is another noteworthy finding. Visceral adiposity is associated with insulin resistance, dyslipidemia, fatty liver, and inflammation^17,18^. A previous intervention study reported reductions in visceral adiposity during Mediterranean diet follow-up, including in the presence of moderate weight loss^4^. Our findings that visceral fat reduction and improved muscle–fat ratio were associated with changes in device-derived metabolic age are consistent with observations from other metabolic populations. For example, Soares-Jr et al. reported differences in type 2 diabetes risk across polycystic ovary syndrome phenotypes despite similar BMI values, highlighting that metabolic risk may vary across phenotypic subgroups^19^. This supports the broader concept that metabolic risk assessment should consider phenotype-related characteristics beyond body weight alone.

Regarding WWI, this analysis showed a significant decrease during follow-up, suggesting an improvement in weight-adjusted central adiposity. WWI has been proposed as an anthropometric index that reflects central adiposity adjusted for body weight and has been associated with cardiometabolic outcomes in previous studies^20^. However, in the adjusted regression model, ΔWWI was not significantly associated with metabolic age change after accounting for weight change and other body composition parameters. Therefore, although WWI improved at the group level, its association with metabolic age change was attenuated when visceral fat, MFR, and protein mass were considered together.

Although mean protein mass did not change significantly at the group level, interindividual changes in BIA-derived protein mass were associated with metabolic age change in Model 2. Because both variables were estimated using the same BIA device, shared measurement or algorithmic components may partly account for this association. More broadly, Mediterranean dietary patterns, which emphasize unsaturated fats and plant-based protein sources, have been associated with favorable metabolic and body composition outcomes^15,21^.

Several limitations should be acknowledged. The retrospective observational design and absence of a control group preclude causal inference; therefore, the observed changes in metabolic age and body composition cannot be attributed definitively to the Mediterranean diet. The observed changes may also have been influenced by regression to the mean, concurrent lifestyle changes, natural temporal variation, or other unmeasured factors. Dietary adherence was not assessed using a validated Mediterranean diet adherence scale, dietary recall, food-frequency questionnaire, or objective nutritional biomarker. In addition, changes in physical activity and medication use were not systematically monitored, and chronic disease status was not incorporated into the analyses. These unmeasured or incompletely measured factors may have confounded the observed associations between changes in body composition and metabolic age. Furthermore, the shared device-derived nature of the outcome and several explanatory variables creates a risk of conceptual and statistical circularity, which limits the interpretation of the observed associations. Biochemical markers such as insulin resistance indices, lipid parameters, inflammatory markers, or hormonal variables were not available, limiting mechanistic interpretation. Several body composition variables were physiologically interrelated, which may have increased multicollinearity. Moreover, because women constituted 82.6% of the sample, the findings may have limited generalizability to men and to populations with a more balanced sex distribution. Finally, the device-specific nature of metabolic age may also limit generalizability across different BIA devices or biological aging measures. Future prospective controlled studies should use validated or objective measures of Mediterranean diet adherence, systematically monitor physical activity and medication changes, and incorporate biochemical or biological aging markers that are independent of the body composition device.

In this retrospective cohort, favorable changes in body composition and Tanita-derived metabolic age were observed during 6-month Mediterranean diet follow-up. Changes in MFR, visceral fat, and protein mass were associated with metabolic age change after adjustment for weight change and baseline covariates, whereas WWI was not significantly associated in the adjusted model. These findings should be interpreted as correlates of a composite device-derived surrogate estimate rather than as evidence that body composition variables independently determine biological aging. Given the uncontrolled design, potential unmeasured confounding, and overlap with the device algorithm, causal conclusions regarding the Mediterranean diet cannot be drawn.

## Data Availability

The datasets generated and/or analyzed during the current study are available from the corresponding author upon reasonable request.
